# Experimental Study on Influence of Trap Parameters on Dielectric Characteristics of Nano-Modified Insulation Pressboard

**DOI:** 10.3390/ma10010090

**Published:** 2017-01-22

**Authors:** Qingguo Chen, Heqian Liu, Minghe Chi, Yonghong Wang, Xinlao Wei

**Affiliations:** Key Laboratory of Engineering Dielectric and its Application, Ministry of Education, Harbin University of Science and Technology, Harbin 150040, China; qgchen@263.net (Q.C.); shotpbs@163.com (M.C.); wyh9195@163.com (Y.W.); weixinlao@163.com (X.W.)

**Keywords:** oil-paper insulation, nanocomposite, dielectric characteristics, trap parameters

## Abstract

In order to study the influence of trap parameters on dielectric characteristics of nano-modified pressboards, pressboards were made using the nano doping method with different nanoparticle components. The dielectric characteristics of the modified pressboards were measured, and the trap parameters were investigated using the thermally stimulated current (TSC) method. The test results indicated that the conductivity initially declined and then rose with the increase of nano-Al_2_O_3_ content, whereas it solely rose with the increase of nano-SiC content. Moreover, the conductivity exhibited nonlinear characteristics with the enhancement of electric field stress at high nanoparticle content. The relative permittivity of modified pressboard declines initially and then rises with the increase of nanoparticle content. In addition, the breakdown strength of modified pressboards exhibited a pattern of incline followed by decline with the increase of nano-Al_2_O_3_ content, while it always declined with the increase of nano-SiC content. The analysis based on the energy band theory on trap parameters of the constructed multi-core model concludes that the nanoparticle components added in pressboard altered both the depth and density of traps. It is therefore concluded that trap parameters have significant influence on the dielectric characteristics of nano-modified insulation pressboard.

## 1. Introduction

As the major equipment used in high voltage direct current (HVDC) transmission systems, the converter transformer plays an important role in connecting the alternating current (AC) network to direct current (DC) network, and its reliability has direct influence on the operational safety of a power system. The oil-paper insulation is widely used in converter transformers and barrier systems as one of the most mature insulation methods. Different from a regular transformer, the converter transformer withstands not only AC voltage, but also AC-superimposed DC voltage and polarity reversal voltage during operations. Additionally, the electric field is frequently concentrated to cause abnormal discharge even breakdown in oil-paper insulation structures [1,2]. Moreover, taking account of the influences of temperature, moisture, space charge and electrical/thermal ageing, the insulation of the convertor transformer is very complicated [3,4,5].

In order to improve the dielectric performance of the insulation pressboard, much work has been done on this particular aspect in recent years [6]. Kamata et al. found that point-to-multi-point (PMP) fiber, nano-montmorillonite (MMT), and nano-SiO_2_ can reduce the relative permittivity of the pressboards [7,8,9]. Liao et al. reported that nano-TiO_2_ and nano-AlN can be used to improve the AC breakdown voltage of pressboard. Also, nano-AlN, nano-ZnO and nano-TiO_2_ can be used to optimize the accumulation and dissipation characteristics of space charge in oil-paper insulation [10,11,12,13]. Chen found that the conductivity of nano-SiC-modified pressboards exhibit obvious nonlinear characteristics at high nano content, which can be used to realize the electric field homogenization in the oil-paper insulation structure under DC and the polarity reversal voltage [14]. Most of these improvements can contribute to the influence of traps. As a typical method to investigate the trap characteristics, the change of thermally stimulated current (TSC) with temperature can be measured through strong electric field injection, rapid freezing and linear heating processes. Finally, the trap parameters can be quantitatively calculated by analyzing the current peak temperature, as well as the shape and size of TSC curves [15,16,17]. Therefore, the nano-Al_2_O_3_ and nano-SiC-modified pressboards were developed by the nano doping method in laboratory. Then the dielectric characteristics of modified pressboard were studied, followed by calculation of trap parameters through TSC method. Further, the effect of trap parameters on dielectric characteristics of the modified pressboard was discussed by using energy band theory with a constructed multi-core model.

## 2. Experiment

### 2.1. Sample Preperation

**Figure 1:**
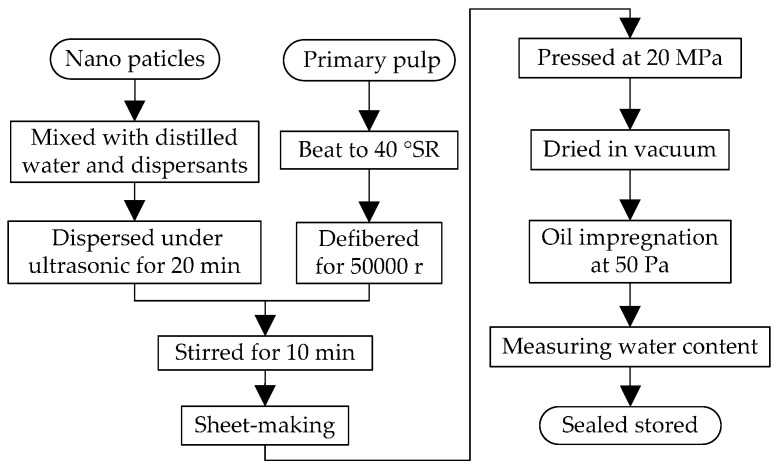
The flow chart of making process of nano-modified pressboard.

The nano-modified pressboards are made of unbleached coniferous kraft pulp, distilled water (μ < 10 S/cm), Al_2_O_3_ nanoparticles (α-Al_2_O_3_, 30 nm) and SiC nanoparticles (β-SiC, 30 nm). According to industrial manufacturing processes of insulation pressboards, the samples are made through six steps i.e., pulping, doping, shaping, compressing, drying and oil impregnating by using the beater, ultrasonic dispersion instrument, standard agitator, handsheet former, curing press, and vacuum drying chamber, as shown in Figure 1, in which SR is the unit of beating degree.

**Figure 2:**
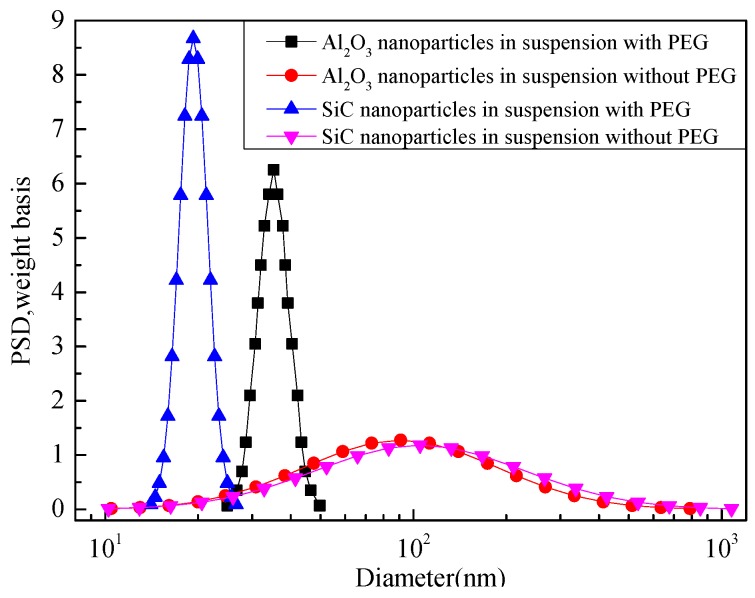
Size distribution of Al_2_O_3_ and SiC nanoparticles in suspension with and without polyethylene glycol (PEG).

Furthermore, polyethylene glycol (PEG) is used for surface modification with the help of space location-obstruct effect to avoid the aggregation of nanoparticles in suspension [18]. After being treated with ultrasonic dispersion for 20 min, the size distribution of nanoparticles is measured by granulometer (Quantachrome Instruments, DT1202). As can be seen from Figure 2, the distribution curves of the untreated Al_2_O_3_ and SiC nanoparticles in suspension have peak values at about 90 nm as the particles are distributed dispersedly, while the nanoparticles treated by PEG are distributed uniformly, whose peak values of the curves are located at about 30 nm and 20 nm. This indicates that it is feasible to reduce the nanoparticle diameter, and the uniformity of the nanoparticle suspension liquid can be maintained by adding PEG. In this process, the mass fraction of nanoparticles in modified pressboards is controlled by changing the quality of the nanoparticle added in the suspension. Moreover, the fine combination with cellulose and the retention quality of nanoparticles is guaranteed by the twining effect because of the long-chain structure of PEG.

**Figure 3:**
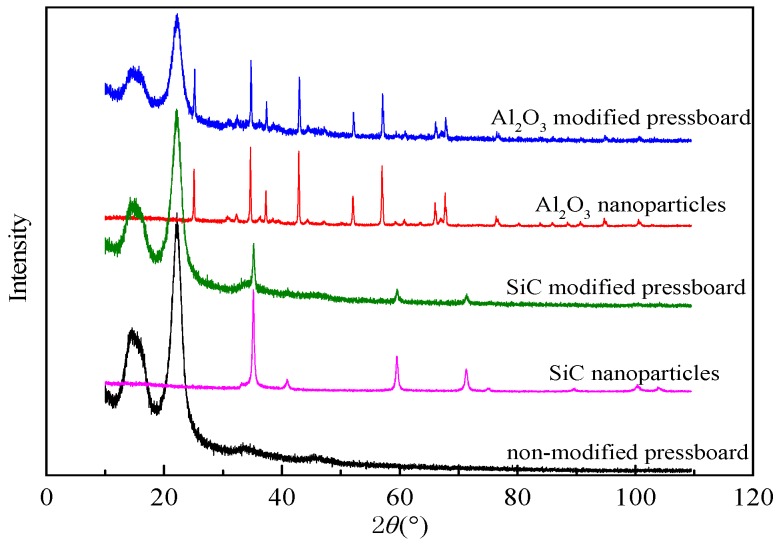
XRD spectra of nanoparticle, non-modified pressboard and modified pressboard.

The X-ray diffraction (XRD) (PANalytical B.V., Almelo, The Netherlands) curves of nanoparticles, non-modified pressboard and modified pressboards are shown in Figure 3.

**Figure 4:**
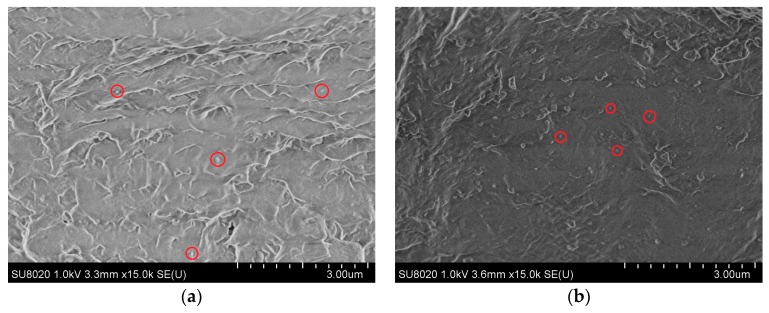

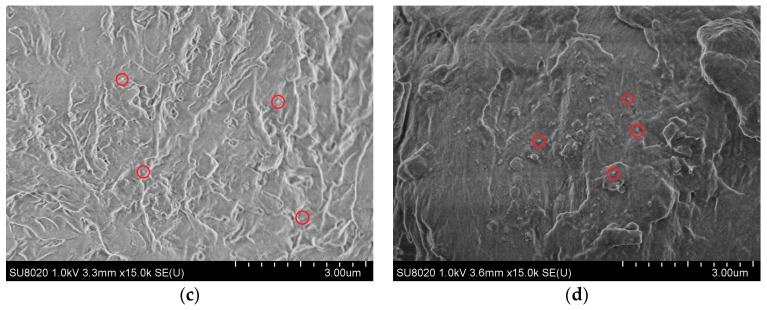
(**a**) SEM micrographs of modified pressboard with 2.5 wt % Al_2_O_3_; (**b**) SEM micrographs of modified pressboard with 7.5 wt % Al_2_O_3_; (**c**) SEM micrographs of modified pressboard with 2.5 wt % SiC; (**d**) SEM micrographs of modified pressboard with 7.5 wt % SiC.

These show that the characteristic peaks in curve of the modified pressboard are identical to these of both non-modified pressboard and nanoparticles. In addition, there is no other characteristic peak, which suggests that the addition of PEG can help controlling the diameter of nanoparticles without introducing by-products. The microstructures of modified pressboards are shown in scanning electron microscopy (SEM) (HITACHI, Tokyo, Japan) micrographs of Figure 4.

Finally, the modified pressboard is obtained with thickness of 0.2 mm and moisture content less than 0.4%. In addition, the tensile strength of pressboard is tested according to the standard ISO 1924-1:1994, ISO 1924-2:1994 and ISO 186:2002, and the results are listed in Table 1. It shows that the tensile strength slightly decreases with the increase of nanoparticle content within acceptable limits.

### 2.2. Measurement System

The conductivity characteristics of modified pressboard were studied by measuring leakage current with the three terminal electrode system, which was connected to a picoammeter. The electrical field stress is applied on the sample by DC high-voltage generators, ranging from 1 kV/mm to 15 kV/mm. Also, the relative permittivity of modified pressboard within 10^−1^ to 10^6^ Hz is measured by the Novocontrol broadband dielectric spectrometer with gold-plating copper electrodes in diameter of 20 mm. In addition, high-voltage generators and plate polar structure in compliance with the standard ASTM-D149 are applied during DC and AC breakdown strength tests. Moreover, the thickness at the breakdown point was measured for calculating, and multiple measuring data was averaged to weaken the influence of data scattering caused by preparation procedures for above tests.

**Figure 5:**
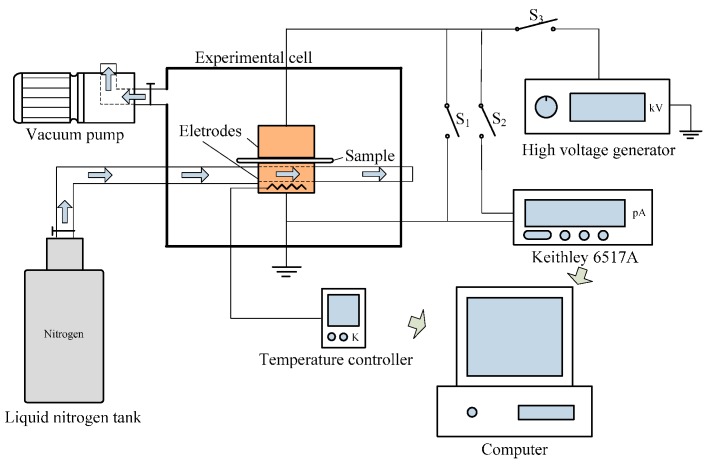
Schematic diagram of thermally stimulated current (TSC) measurement system.

**Figure 6:**
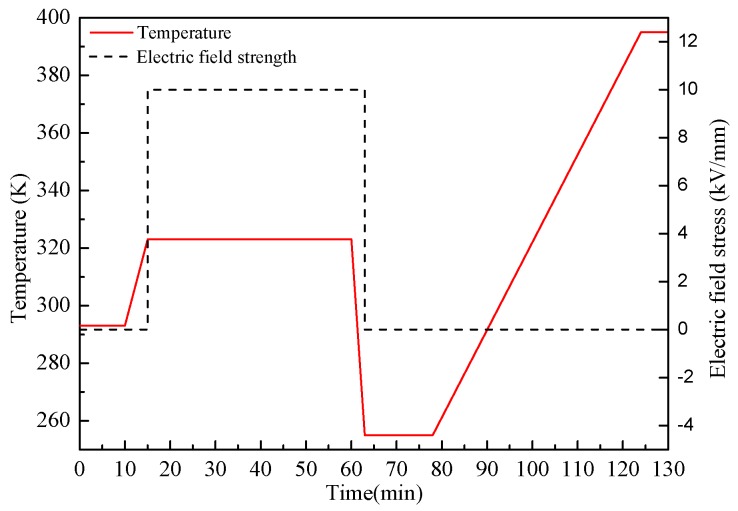
Temperature and electric field stress applied in experiment versus time.

To characterize the trap parameters of nano-modified pressboards, the TSC curves are obtained by Keithley 6517 A, cooperating with heating and cooling system, vacuum equipment and a DC high voltage generator. The schematic diagram of TSC measurement system and procedure are shown in Figure 5 and Figure 6.

## 3. Results and Discussion

### 3.1. Conductivity Characteristics of Modified Pressboard

**Figure 7:**
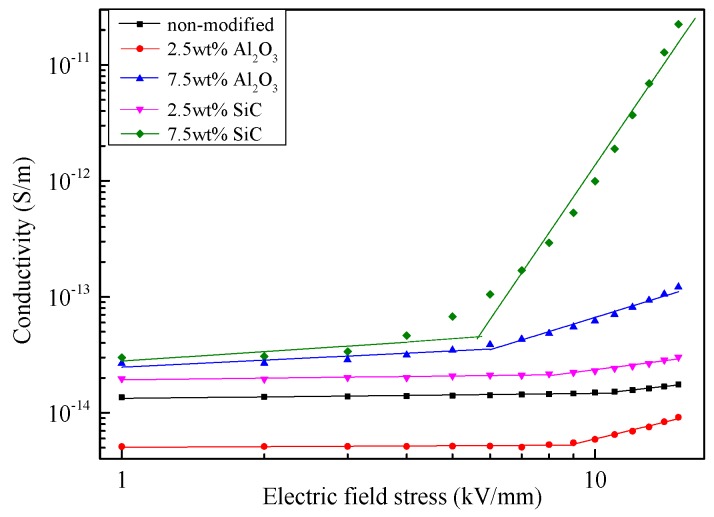
Conductivity versus *E* curves of pressboards with different nanoparticle components.

The relationships between conductivity (γ) and electric field stress (*E*) of pressboards with different nanoparticle components are shown in Figure 7. It shows that the conductivities of modified pressboards are higher than that of non-modified pressboard, except the nano-Al_2_O_3_-modified pressboard at 2.5 wt %. For the same nano doping material, the conductivity of modified pressboard rises as the content increases. Meanwhile, the conductivity of nano-SiC-modified pressboard is higher than that of nano-Al_2_O_3-_modified pressboard at same content. Moreover, nano-SiC-modified pressboards show more obvious nonlinear characteristics under high electric field strength.

### 3.2. Relative Permittivity Characteristics of Modified Pressboard

**Figure 8:**
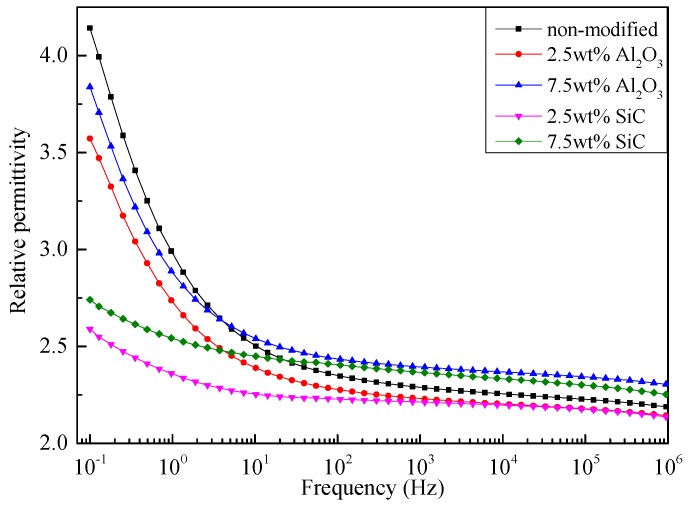
ε_r_ versus frequency curves of pressboards with different nanoparticle components.

The relationships between relative permittivity (ε_r_) and frequency of pressboards with different nanoparticle components are shown in Figure 8. The ε_r_ values of the modified pressboards with the same nano doping material express a trend of decline and then incline with the increase of nanoparticle content. In detail, ε_r_ alters slowly with the decrease of frequency at high frequency area, while it increases rapidly at low frequency area.

### 3.3. Breakdown Strength Characteristics of Modified Pressboard

**Figure 9:**
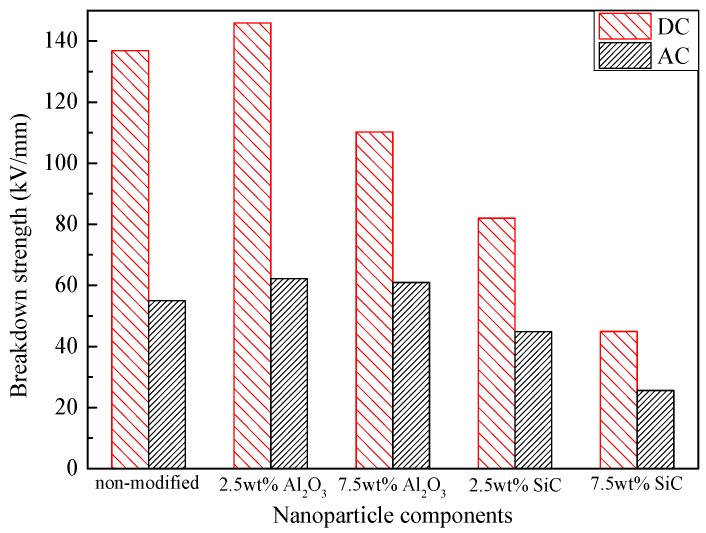
Breakdown strength histogram of pressboards with different nanoparticle components.

The relationships between electric breakdown strength and nanoparticle components are shown in Figure 9. The electric breakdown strength of nano-Al_2_O_3_-modified pressboard rises firstly and falls afterwards as the nanoparticle content increases, and it reaches the peak value at 2.5 wt %. Specifically, the breakdown strength of the nano-SiC-modified pressboard is lower than that of the nano-Al_2_O_3_-modified pressboard at the same content, and it decreases further as the content increases.

### 3.4. TSC Test Results of Modified Pressboard

**Figure 10:**
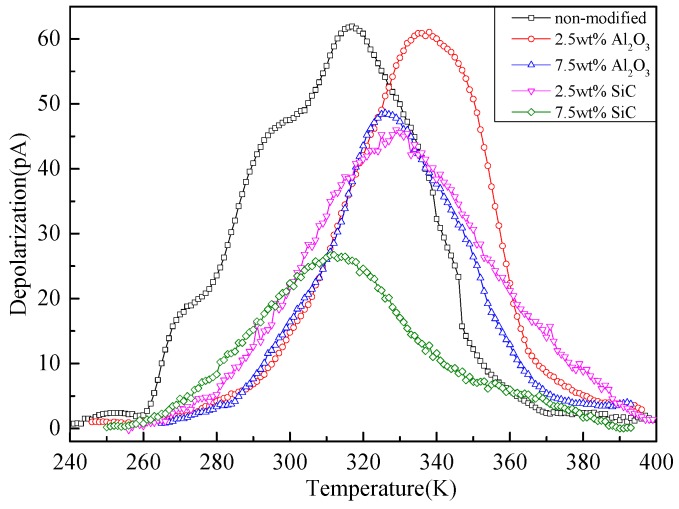
TSC curves of pressboards with different nano doping components.

To represent the trap parameters of modified pressboard, the TSC test is carried out by the mentioned equipment, and the curves of pressboards with different nanoparticle components are shown in Figure 10.

### 3.5. Discussion

**Figure 11:**
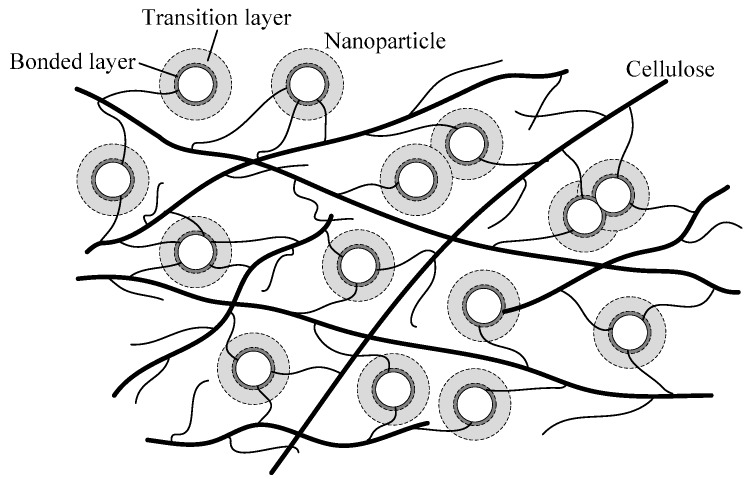
Distribution model of nanoparticles in modified pressboard.

According to the research of Tanaka, the interface around the nanoparticles consists of the bonded layer and transition layer, which provides deep and shallow traps separately [19,20]. Considering the loose and porous structure of pressboard, the nanoparticles mainly exist in free volume of cellulose matrix. The distribution model of nanoparticles can be represented as Figure 11.

**Figure 12:**
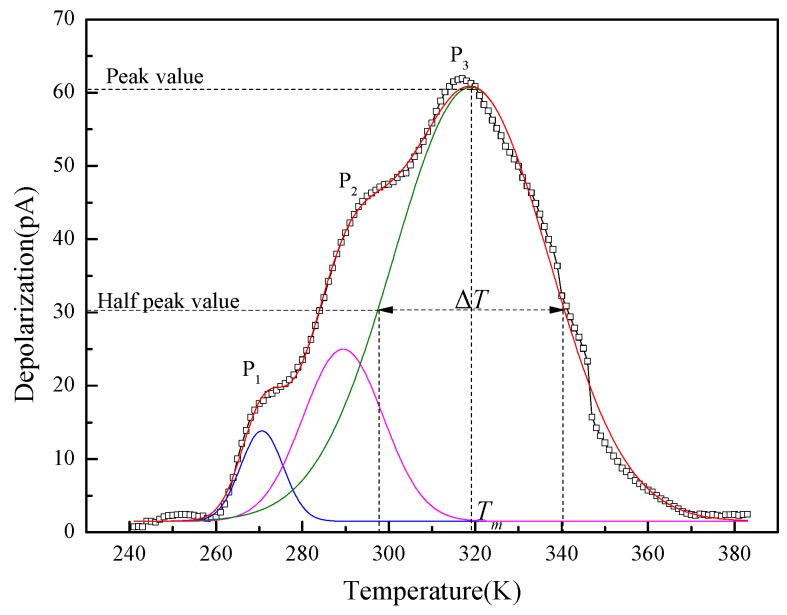
TSC curves of non-modified pressboard.

As trap is essentially the localized state in the forbidden band which has constraint on ions, it can be formed not only by branches and end groups of cellulose, but also the lattice defects of nanoparticles in the modified pressboard system [21]. Thus, the doping of nanoparticles can introduce a new localized state in the system, which results in the change of trap depth and density [22]. The TSC curve of the non-modified pressboard analyzed by the Gauss multi-peak fitting is shown in Figure 12.

In contrast to TSC curves of modified pressboards with single peak, three peaks can be stripped from the TSC curve of non-modified pressboard. The temperatures correspond to P_1_ and P_2_ is much lower than that of P_3_, which reveals the depth relationship of trap level correspond to each peaks. According to the research of Ieda, the release of carriers from traps is related to the molecular motion [23]. Also, the disappearance of the P_1_ and P_2_ peak indicates the enhancement of restriction to branches by interface. Due to the high molecular weight of cellulose, the dipole orientation polarization is mainly caused by the rotation of the polar groups. Thus, ε_r_ value decreases after modification, while for the modified pressboard at 7.5 wt %, ε_r_ increases slightly as a result of the interfacial polarization.

The quantity of trap charge can be calculated through Equation (1) by analyzing the TSC curve in Figure 10:

(1) $$Q_{TSC}=\int_{t_{2}}^{t_{1}}I\left(t\right)dt=\frac{60}{\beta }\int_{T_{2}}^{T_{1}}I\left(T\right)dt,$$

where *I*(*T*) is the TSC current value, *T*_1_ and *T*_2_ is the initial and end temperature respectively, and β is the temperature rise rate, whose value is 3 K/min.

Meanwhile, the trap level can be calculated by Equation (2):

(2) $$E=\frac{2.47T_{m}{}^{2}k}{∆T},$$

where *T_m_* is the temperature corresponding to the peak value of the stimulated current, ∆*T* is the temperature difference between the two half peak values, and *k* is Boltzmann constant [24]. For the non-modified pressboard, the relative shallow traps play an auxiliary conduction role in carrier transport, and only the parameter of P_3_ peak is taken into account. The trap parameters of pressboards with different nanoparticle component are shown in Table 2.

It can be seen that the quantity of the trap charge and the trap level generally shows a trend of incline and then decline as the nano-Al_2_O_3_ content increases, while they decline further as the increase of nano-SiC content. Additionally, the quantity of trap charge of the nano-SiC-modified pressboard at 2.5 wt % is higher than that of the nano-Al_2_O_3_-modified pressboard at 7.5 wt %.

For trap density, the nanoparticles were distributed uniformly at low nanoparticle content, so the interfacial volume increases obviously, and more traps are introduced into the system. However, as the nanoparticle content increases, the partial aggregation of nanoparticles intensifies in actual distribution. Thus, the interfacial volume decreases because of the overlapping of the transition layer, which provides shallow traps. Moreover, the probability of contact between nanoparticles and end groups of cellulose increases because of the heterogeneous nucleation effect, which will consume a number of traps. As a result, the trap density is reduced.

**Figure 13:**
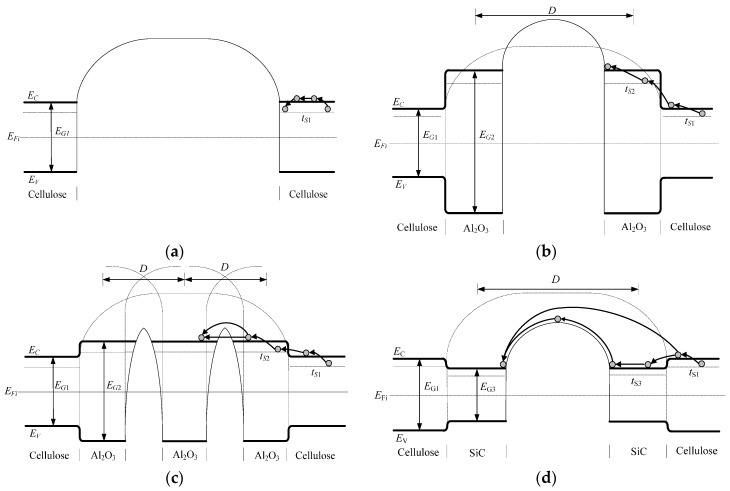

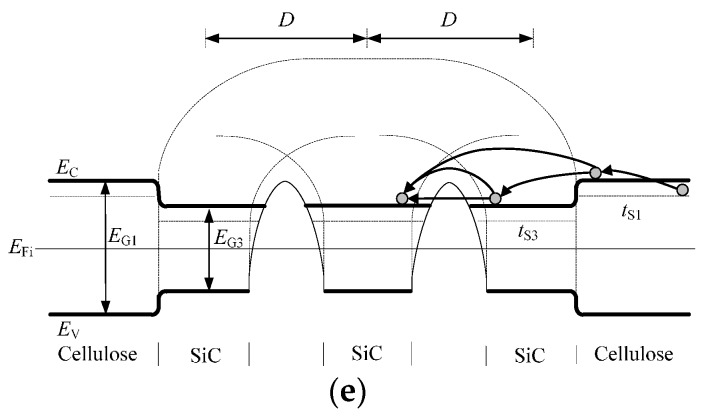
(**a**) Energy band structure of non-modified pressboard; (**b**) Energy band structure of modified pressboard with 2.5 wt % Al_2_O_3_; (**c**) Energy band structure of modified pressboard with 7.5 wt % Al_2_O_3_; (**d**) Energy band structure of modified pressboard with 2.5 wt % SiC; (**e**) Energy band structure of modified pressboard with 7.5 wt % SiC. And *D* is the separation distance between neighbor nanoparticles, *E*_C_ is the conduction band, *E*_V_ is the valence band, *E*_Fi_ is the Fermi level, *E*_G1_ is the width of forbidden band of cellulose, *E*_G2_ is the width of forbidden band of Al_2_O_3_ nanoparticles, *E*_G3_ is the width of forbidden band of SiC nanoparticles, while *t*_s1_, *t*_s2_, *t*_s3_ are the trap energy levels of cellulose, Al_2_O_3_ nanoparticles, and SiC nanoparticles separately.

For trap depth, it should not be simply described as the energy difference between the bottom of the polymer molecule conduction band and the trap energy level. Rather, it is supposed to represent the required energy for carriers to jump from the trap energy level to the specific energy level in which they can participate in electric conduction in nanocomposite [25]. As Figure 13 shows, the potential barrier between neighbor crystalline regions is too high and too wide for electrons to pass through, which causes the low conductivity in the non-modified pressboard. In the nano-Al_2_O_3_-modified pressboard with low content, the potential barrier formed by the interaction of nanoparticles and cellulose is higher than that of neat cellulose because of the wide forbidden band of Al_2_O_3_. However, as the nanoparticle content increases, the separation distance between neighbor nanoparticles becomes closer, and the nanoparticles are affected by the potential field from each other, the degeneracy of energy level decreases [26]. Therefore, the width of the permissible band increases, and the width of the forbidden band decreases accordingly. As a result, the electrons can reach the conduction band of Al_2_O_3_ nanoparticles by jumping continuously with the help of the traps provided by the Al_2_O_3_ nanoparticles. Then, the electrons can get over or go through the potential barrier, and the change of conductive mechanism causes the non-linearity of conductivity of the pressboard with high nanoparticle content. Similarly, given the narrow forbidden band of SiC, the potential barrier formed by the interaction between nanoparticles and cellulose is lower than that of the non-modified pressboard. Under such a condition, electrons are able to jump over the potential barrier directly from the cellulose conduction band. Since the conductive mechanism is not changed, the degree of non-linear characteristic does not alter much, and it is lower than that of the nano-Al_2_O_3_-modified pressboard at 7.5 wt %. As the nanoparticle content increases, the SiC nanoparticles have more obvious effect on lowering the potential barrier than Al_2_O_3_ nanoparticles, which results in higher conductivity and degree of non-linear characteristic. The model of band structures and transition paths of electrons in modified pressboard with different nanoparticle components are shown in Figure 13.

According to Figure 12, the transition energy rises at first and then falls with the increment of nano-Al_2_O_3_ content, whereas it decreases strictly as the nano-SiC content increases.

To analyse the non-linear characteristic of dielectric material, the relationship between the conductivity of the modified pressboard and electric field stress can be expressed as:

(3) $$\gamma =AE^{\beta },$$

By logarithmic transformation of Equation (3), there is:

(4) logγ=logA+βlogE,

where *A* is a constant related to material properties, and β is the non-linear conductive coefficient. Thus, there is a linear relationship between lg γ and lg *E* in log-log coordinate, where the slope of the changing curve β represents the degree of nonlinear characteristic [27]. By using linear fit in two segments, the threshold electric field at which the non-linear conductive coefficient changes can be defined as *E*_cr_, and the non-linear conductive coefficient β_1_ (below *E*_cr_) and β_2_ (upon *E*_cr_) are shown in Table 3.

As shown in Table 3, when the nanoparticle content of a certain nano doping material increases, the *E*_cr_ of the modified pressboard decreases, and the non-linear conductive coefficient increases. In addition, the *E*_cr_ of the nano-SiC-modified pressboard is lower than that of the nano-Al_2_O_3_-modified pressboard at the same nanoparticle content, and its non-linear conductive coefficient is generally higher, especially at high nanoparticle content, which is in agreement with the theoretical analysis by modeling.

**Figure 14:**
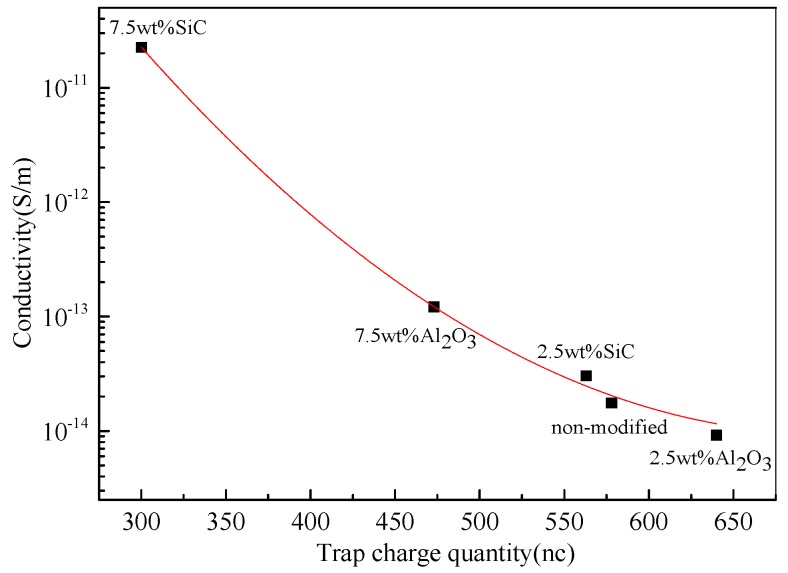
Relationship between conductivity and trap charge quantity.

Moreover, the research shows that the trap parameters have a great influence on charge storage and transportation, as well as the electrical performance such as conductive and breakdown characteristics in polymer and nanocomposite [20]. The relationship between trap charge quantity and conductivity is shown in Figure 14. The conductivity decreases with the increase of trap charge quantity. This is because the increase of charge trap sites in nanocomposite can reduce the change mobility and energy of the charge carriers, which contributes to the decrease of conductivity [21].

**Figure 15:**
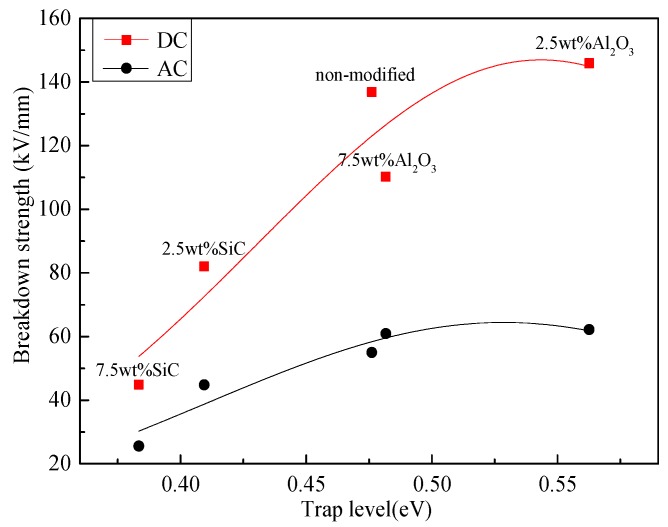
Relationship between breakdown strength and trap level.

Furthermore, relationship between breakdown strength and trip level is shown in Figure 15. It shows that the breakdown strength increases with the increase of trap level. As mentioned earlier, as the trap level increases, more energy is consumed for carriers to get into and out of the traps, the mean free path of carriers can be shortened during the process, which makes it more difficult to form the effective carriers [28]. As a result, the breakdown strength is enhanced. Overall, the variation of the trap parameters is one of the main reasons for the change of the dielectric characteristics of the modified pressboard.

## 4. Conclusions

Based on the experimental study and modeling analysis on dielectric characteristics and trap parameters of nano-modified pressboards, the following conclusions have been drawn:

(1) The depth and density of traps of pressboard can be altered by nano-modification. Both of them rise initially and then decline with the increase of nanoparticle content.
(2) The forbidden bandwidth of the nanoparticle can significantly influence the trap depth. It decreases with the narrowing of the forbidden bandwidth, and the conductivity exhibits more obviously nonlinear characteristics due to variation of energy band structure.
(3) The conductivity decreases with the increase of trap charge quantity, and the breakdown strength increases with the increase of trap level, which indicates that the trap parameters have significant influence on dielectric characteristics.

## Figures and Tables

**Table 1:** Tensile strength of pressboards with different nanoparticle component.

Nanoparticle Components	Non-Modified	2.5 wt % Al_2_O_3_	7.5 wt % Al_2_O_3_	2.5 wt % SiC	7.5 wt % SiC
Tensile strength (kN/m)	6.79	6.43	5.91	6.25	5.76

**Table 2:** Trap parameters of pressboards with different nanoparticle component.

Nanoparticle Components	Peak Current Value (pA)	Peak Value Temperature (K)	Trap Charge Quantity (nC)	Trap Level (eV)
non-modified	60	318	578.8	0.4761
2.5 wt % Al_2_O_3_	61	339	640.3	0.5626
7.5 wt % Al_2_O_3_	48	326	473.5	0.4817
2.5 wt % SiC	46	331	563.6	0.4094
7.5 wt % SiC	27	310	300.2	0.3834

**Table 3:** Non-linear parameters of modified pressboards.

Nanoparticle Components	*E*_cr_ (kV/mm)	β_1_	β_2_
Non-modified	11	0.0435	0.4507
2.5 wt % Al_2_O_3_	9	0.0194	0.9904
7.5 wt % Al_2_O_3_	6	0.2001	1.2561
2.5 wt % SiC	8	0.0487	0.5280
7.5 wt % SiC	5	0.2696	5.9972
